# Alcohol and hepatitis virus-dysregulated lncRNAs as potential biomarkers for hepatocellular carcinoma

**DOI:** 10.18632/oncotarget.22921

**Published:** 2017-12-05

**Authors:** Hao Zheng, Pinxue Li, James G. Kwok, Avinaash Korrapati, Wei Tse Li, Yuanhao Qu, Xiao Qi Wang, Tatiana Kisseleva, Jessica Wang-Rodriguez, Weg M. Ongkeko

**Affiliations:** ^1^ Department of Surgery, University of California, San Diego, La Jolla, California, USA; ^2^ Department of Surgery, The University of Hong Kong, Pokfulam, Hong Kong, China; ^3^ Veterans Administration Medical Center and Department of Pathology, University of California, San Diego, La Jolla, California, USA

**Keywords:** lncRNA, hepatocellular carcinoma, alcohol, hepatitis

## Abstract

Hepatocellular carcinoma (HCC) is one of the leading causes of cancer-related deaths because of frequent late detection and poor therapeutic outcomes, necessitating the need to identify effective biomarkers for early diagnosis and new therapeutic targets for effective treatment. Long noncoding RNAs (lncRNAs) have emerged as promising molecular markers for diagnosis and treatment. Through analysis of patient samples from The Cancer Genome Atlas database, we identified putative lncRNAs dysregulated in HCC and by its risk factors, hepatitis infection and alcohol consumption. We identified 184 lncRNAs dysregulated in HCC tumors versus paired normal samples, 53 lncRNAs dysregulated in alcohol-drinking patients with hepatitis B, and 5, 456 lncRNAs dysregulated in patients with hepatitis infection. A panel of these candidate lncRNAs’ expressions correlated significantly with patient survival, clinical variables, and known genomic alteration in HCC. Two most significantly dysregulated lncRNAs in our computational analysis, lnc-CFP-1:1 and lnc-CD164L2-1:1, were validated *in vitro* to be dysregulated by alcohol. Our findings suggest that lncRNAs dysregulated by different etiologies of HCC serve as potential disease markers and can be further investigated to develop personalized prevention, diagnosis, and treatment strategies.

## INTRODUCTION

Hepatocellular carcinoma (HCC) is the most common class of liver cancer, accounting for 70–90% of primary liver cancer cases [1]. Because of limitations in diagnostic methods, HCC is often diagnosed late, when intrahepatic and extrahepatic metastasis are likely to have already occurred, leading to poor clinical outcome and therapeutic response [1, 2]. HCC causes 750, 000 death annually worldwide, the second highest total mortality out of all human cancers [3]. The five-year survival rate for HCC has remained below 20% [4]. Therefore, there is an urgent need for the discovery of biomarkers that can allow for early diagnosis of HCC and prediction of metastasis risk.

Long noncoding RNAs (lncRNAs) are noncoding RNAs over 200 bases in length that serve a variety of roles in regulating protein levels and gene expression [1]. LncRNAs have been extensively documented as important regulatory molecules involved in tumorigenesis [5]. A number of lncRNAs were revealed to have significant functions in the pathogenesis of HCC and could serve either tumor suppressing or oncogenic roles. Active pathological mechanisms in HCC such as the Wnt signaling pathway, the STAT3 signaling pathway, and epithelial-to-mesenchymal transition (EMT) were shown to be regulated by lncRNAs such as DANCR, ATB, and MALAT1, respectively [6–8]. Other lncRNAs involved in HCC include HOTAIR (HOX transcript antisense RNA) and HULC (Highly Upregulated in Liver Cancer), which are also both involved in multiple cancers beside HCC [3, 9]. Because of their extensive involvement in tumorigenesis, lncRNAs are promising candidates as biomarkers for prediction of prognosis in HCC.

Important risk factors in the development of HCC are alcohol consumption and the hepatitis virus. An estimated half of HCC patients have an hepatitis B virus (HBV) infection, while thirty to forty percent of HBV patients ultimately develop HCC [10, 11]. Hepatitis C virus (HCV) infection causes an estimated seventeen-fold increase in the risk of developing HCC compared to non-hepatitis individuals [12]. For people with heavy alcohol consumption, ten to thirty percent develop alcoholic steatohepatitis, while ten to twenty percent develop liver cirrhosis [13]. Both alcoholic steatohepatitis, a type of fatty liver disease, and liver cirrhosis can lead to development of HCC. Because HCC is a heterogeneous disease with various prognostic factors and prognostic outcomes, the risk factors involved in the development of HCC for specific patients must be considered in order to develop personalized diagnostic and treatment methods.

Most studies investigating lncRNAs involved in HCC were limited by the small size of patient cohorts and the lack of specific HCC etiology focus, with many studies comparing only tumor samples with normal samples [1]. While there have been studies exploring differential gene expression for coding genes in HCC caused by different risk facts, to the best of our knowledge, no study has compared the role of lncRNAs in HBV/HCV- related HCC to their role in alcohol-related HCC. We downloaded RNA-sequencing data for 222 HCC patients from The Cancer Genome Atlas (TCGA) database to obtain a substantial cohort for analysis. We then analyzed lncRNA expression levels of patient normal samples versus that of three tumor sample cohorts, based on HBV infection, HCV infection, and history of alcohol consumption. Finally, the expressions of selected lncRNAs were verified *in vitro*.

## RESULTS

### Identification of alcohol and hepatitis-dysregulated lncRNAs

**Figure 1:**
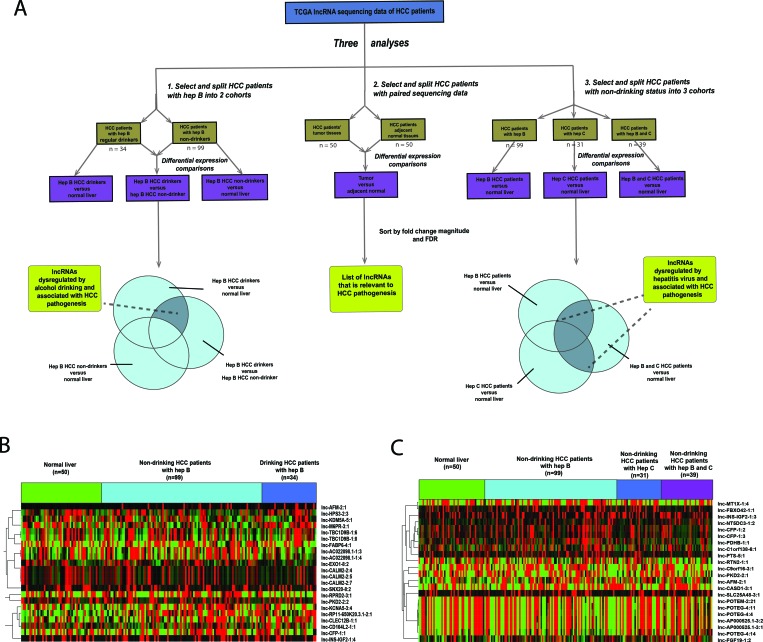
Identification of alcohol and hepatitis-dysregulated lncRNAs (**A**) Schematic illustrating the analysis approach used to identify alcohol and hepatitis-dysregulated lncRNA candidates (*p* < 0.05, FDR < 0.05). (**B**–**C**) Heatmap depicting normalized expression levels (in counts-per-million) of the 22 lncRNAs transcripts with the largest magnitude of dysregulation in (B) HBV+ HCV− drinker versus HBV+ HCV− nondrinker and (C) HBV+ HCV+ nondrinker versus normal liver.

Clinical and RNA-sequencing data for 222 HCC patients and 50 normal liver tissues were sorted into four cohorts based on their clinical history on viral hepatitis infection and alcohol consumption: (1) HBV+ HCV− drinker (*n* = 34); (2) HBV+ HCV− non-drinker (*n* = 109); (3) HBV− HCV+ nondrinker (*n* = 32); and (4) HBV+ HCV+ nondrinker (*n* = 47). To identify lncRNAs dysregulated by alcohol in the context of HBV, three differential expression analyses were performed using the Bioconductor package edgeR: (a) HBV+ HCV− drinker versus normal liver; (b) HBV+ HCV− non-drinker versus normal liver; and (c) HBV+ HCV− drinker versus HBV+ HCV− non-drinker (Figure 1A). 53 lncRNA transcripts were found to be significantly dysregulated due to alcohol in the context of HBV (FDR < 0.05) (Figure 1B) and the top 10 most dysregulated lncRNAs due to alcohol use are shown in Table 1. To identify lncRNAs dysregulated by hepatitis virus, three differential expression analyses were performed: (d) HBV+ HCV- non-drinker versus normal; (e) HBV− HCV+ non-drinker versus normal liver; and (f) HBV+ HCV+ non-drinker versus normal liver. 12,328, 20,873 and 19,194 lncRNA transcripts were differentially expressed in patients with HBV, HCV, and both HBV and HCV, respectively, compared to normal liver. 5,456 lncRNA transcripts were found to be commonly dysregulated by HBV and HCV in all three comparisons above (FDR < 0.05) (Figure 1C). The top 10 most dysregulated lncRNAs due to hepatitis virus are shown in Table 2. In addition, a pairwise differential expression analysis was performed on 50 tumor and adjacent normal pairs. 184 lncRNAs were found to be significantly dysregulated (FDR < 0.05) in HCC tumors compared to adjacent normal, 32 of which were implicated in the previous alcohol and/or hepatitis analyses. The top 10 most dysregulated lncRNAs in tumor samples are shown in Table 3.

**Table 1:** Significantly dysregulated lncRNAs in alcohol comparisons for HBV+ patients

	HBV+ drinker versus HBV+ nondrinker				HBV+ drinker versus normal liver			
	Fold change	logCPM	P value	FDR	Fold change	logCPM	P value	FDR
**lnc-AFM-2:1**	4.80	8.74	1.3E-04	3.3E-02	120.55	8.62	1.1E-21	5.3E-20
**lnc-HPS3-2:3**	4.29	1.96	1.6E-09	3.9E-06	2.27	2.48	9.1E-04	2.0E-03
**lnc-TBC1D9B-1:6**	3.06	7.28	3.6E-07	5.4E-04	12.35	7.01	2.0E-25	1.8E-23
**lnc-TBC1D9B-1:8**	2.74	7.89	2.0E-06	2.1E-03	9.31	7.59	1.4E-22	8.0E-21
**lnc-M6PR-3:1**	2.26	2.88	1.9E-04	4.1E-02	6.53	2.44	3.7E-20	1.4E-18
**lnc-AC022098.1-1:3**	−2.50	3.59	1.0E-05	6.4E-03	1.76	1.93	4.7E-06	1.6E-05
**lnc-EXO1-8:2**	−2.58	1.79	3.0E-05	1.4E-02	1.33	0.31	1.5E-03	3.2E-03
**lnc-AC022098.1-1:4**	−2.77	3.23	1.9E-05	9.4E-03	1.90	1.39	4.5E-06	1.5E-05
**lnc-CALM2-2:4**	−2.90	2.85	4.8E-05	1.7E-02	1.48	1.11	5.7E-04	1.3E-03
**lnc-CALM2-2:5**	−3.00	2.83	3.5E-05	1.5E-02	1.67	0.97	1.7E-05	5.2E-05

**Table 2:** Significantly dysregulated lncRNAs in hepatitis comparisons

	HBV+ HCV- HCC versus normal liver				HBV- HCV+ HCC versus normal liver				HBV+ HCV+ HCC versus normal liver			
	Fold change	logCPM	*P* value	FDR	Fold change	logCPM	*P* value	FDR	Fold change	logCPM	*P* value	FDR
**lnc-AFM-2:1**	20.14	6.75	4.49E-13	2.01E-12	5.09	4.39	3.56E-06	1.25E-05	17.73	6.24	4.79E-13	4.95E-12
**lnc-C9orf16-3:1**	22.05	5.23	3.93E-21	4.43E-20	26.71	4.87	3.37E-23	2.28E-21	26.02	5.18	1.20E-21	4.30E-20
**lnc-CASD1-3:1**	40.16	7.23	4.67E-19	4.08E-18	23.03	5.79	3.96E-16	8.37E-15	36.30	6.77	1.11E-22	4.51E-21
**lnc-FBXO42-1:1**	–4.70	3.05	5.63E-11	2.00E-10	–4.34	3.76	1.92E-08	1.07E-07	–13.40	3.57	1.56E-26	1.05E-24
**lnc-INS-IGF2-1:3**	–6.76	3.50	1.11E-11	4.25E-11	–6.59	4.20	1.00E-07	4.89E-07	–12.16	4.06	1.57E-14	2.04E-13
**lnc-MT1X-1:4**	–5.29	8.47	2.64E-12	1.08E-11	–3.63	9.26	2.22E-06	8.15E-06	–13.09	9.05	5.67E-23	2.37E-21
**lnc-NT5DC3-1:2**	–12.05	2.76	4.02E-87	1.22E-82	–12.30	3.55	5.68E-32	1.39E-29	–11.81	3.49	1.75E-38	6.62E-36
**lnc-PKD2-2:1**	22.55	5.48	1.02E-16	6.84E-16	13.79	4.17	1.59E-17	4.17E-16	20.68	5.05	1.52E-20	4.68E-19
**lnc-POTEM-2:21**	13.95	3.80	1.12E-34	6.20E-33	13.03	3.13	7.15E-36	2.83E-33	16.44	3.78	6.59E-49	1.51E-45
**lnc-SLC25A48-3:1**	–6.90	3.75	8.34E-14	4.05E-13	–9.83	4.43	1.14E-15	2.20E-14	–16.38	4.32	1.14E-22	4.63E-21

**Table 3:** Significantly dysregulated lncRNAs in paired tumor and adjacent normal analysis

Pairwise tumor versus adjacent normal				
	Fold change	logCPM	*P* value	FDR
**lnc-AKR1B10-1:1**	14.17	7.42	9.48E-10	6.80E-09
**lnc-ASPM-1:4**	14.10	2.13	2.04E-60	3.85E-57
**lnc-ASPM-1:5**	14.10	2.13	2.04E-60	3.85E-57
**lnc-BEST4-1:1**	11.69	2.68	5.71E-40	2.46E-37
**lnc-FABP6-4:1**	11.03	3.69	8.75E-27	9.86E-25
**lnc-CXCL12-4:2**	−16.08	2.47	1.08E-40	5.04E-38
**lnc-TARS2-1:2**	−16.10	2.18	1.94E-44	1.36E-41
**lnc-SEC22C-1:4**	−16.15	1.82	6.77E-41	3.24E-38
**lnc-EIF2AK3-4:9**	−16.25	1.68	5.69E-15	1.02E-13
**lnc-SEC22C-1:12**	−16.27	1.53	1.75E-41	9.11E-39

### Identification of dysregulated lncRNAs correlated to patient survival and other clinical outcomes

**Figure 2:**
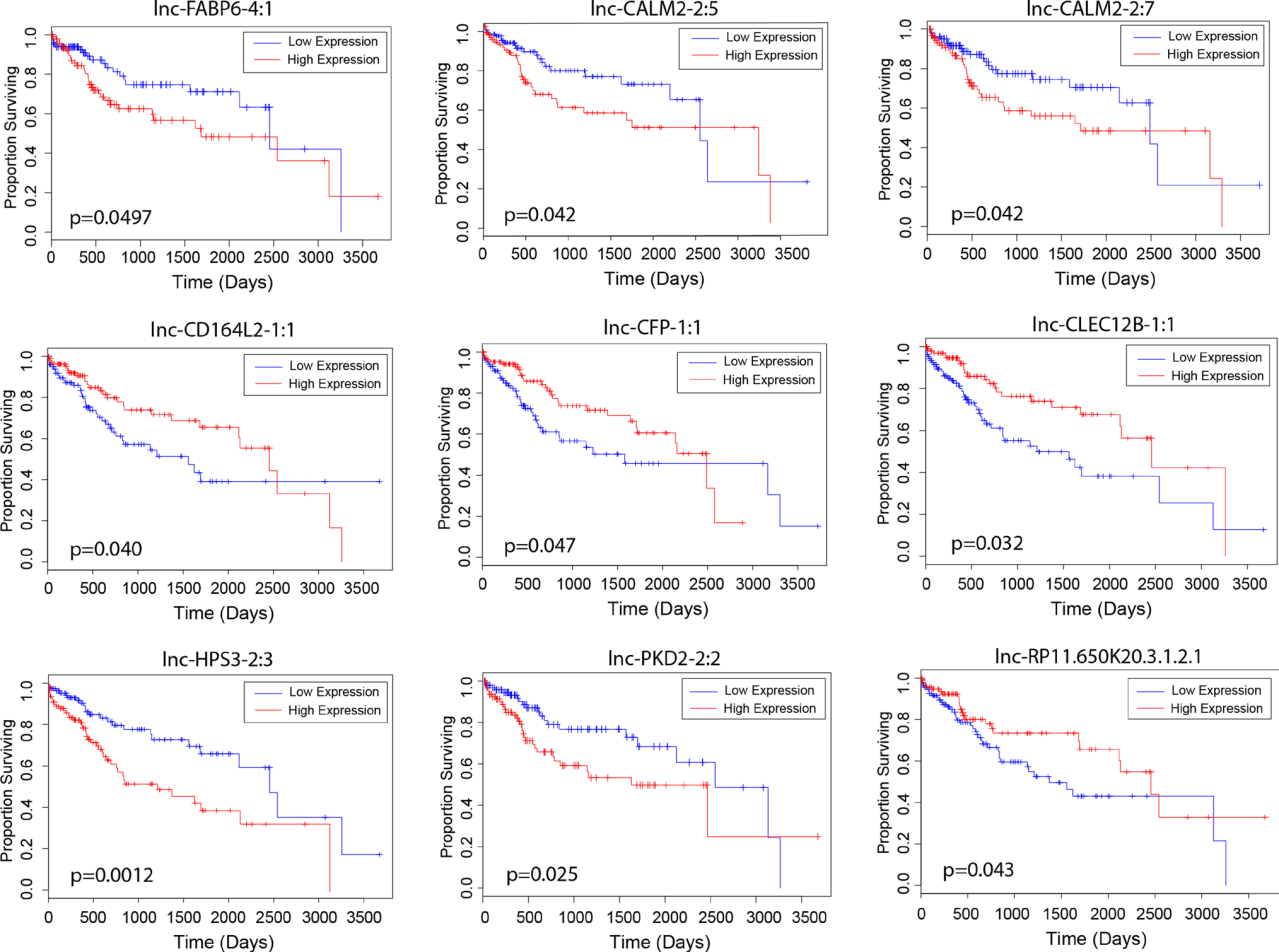
Association of lncRNA expression with patient survival Kaplan-Meier curves depicting survival outcomes based on relative high and low expression of candidate lncRNAs proposed to be dysregulated by alcohol and/or viral hepatitis (Kaplan-Meier, *p* < 0.05).

To explore the clinical significance of lncRNAs identified in the differential expression analyses, we examined the relationship between their expression levels and overall patient survival. Modeling lncRNA expression as a binary variable (high/low), we found that relative high expression of five unregulated lncRNAs (lnc-FABP6-4:1, lnc-CALM2-2:5, lnc-CALM2-2:7, lnc-HPS3-2:3 and lnc-PKD2-2:2) and four downregulated lncRNAs (lnc-CD164L2-1:1, lnc-CFP-1:1, lnc-CLEC12B-1:1 and lnc-RP11.650K20.3.1.2.1) significantly associated with poor patient survival (*p* < 0.05) (Figure 2). Further, we correlated the expression of these genes to key clinical outcomes such as vital status, tumor grade and pathological tumor stage. Three lncRNAs (lnc-CLEC12B-1:1, lnc-HPS3-2:3 and lnc-RP11.650K20.3.1.2.1) significantly correlated with patient vital status (*p* < 0.05). Notably, stage-dependent correlation was observed between the expression level of two lncRNAs, lnc-FABP6-4:1 and lnc-CD164L2-1:1, and tumor grade, a grading system that characterizes level of differentiation of malignant cancer cells. Lastly, stage-dependent correlation was also found between lnc-CFP-1:1 and pathologic tumor stage, a staging system describing the development of liver cancer in terms of pathology.

### Association of dysregulated lncRNAs with known HCC genomic alterations

Numerous genomic alternations, including genetic mutations and DNA copy number variations, have been reported in HCC and have been associated with liver cancer development, progression and metastasis [14, 15]. To explore the potential functions of lncRNAs in relationship to canonical HCC driver events, we utilized Wilcoxon rank-sum testing to identify correlations between lncRNA expression and tumor mutational status or copy number variation. We focused our analysis on the 10 most frequently mutated genes in HCCs, as determined by Debuire *et al.* [16]. We found nine lncRNAs to be significantly associated with mutation status (*p* < 0.05) (Figure 4). Notably, four lncRNAs (lnc-RPRD2-3:1, lnc-KCNA5-3:4, lnc-CFP-1:1 and lnc-CD164L2-1:1) correlated strongly with the mutation status of IGF2R, a tumor suppressor commonly mutated in human liver and breast cancer. lnc-HPS3-2:3 expression significantly correlated with incidence of mutated CTNNB1 (*p* = 0.020), a well-studied proto-oncogene. Additionally, pairwise analysis between lncRNA expression and incidence of copy number variations revealed widespread correlations. lnc-FABP6-4:1, an lncRNA upregulated over 11 fold in HCC tumor compared to normal liver (Table 2), was found to have extensive correlation with copy number variations of 16 locations on chromosomes 4, 8, 9, 13, 14, 16, 17 and 19 (Figure 5A). lnc-AFM-2:1, another upregulated lncRNA in HCC patients (Table 1), correlated strongly with copy number variations of two locations on chromosome 8 (*p* < 0.01) (Figure 5B). Moreover, three lncRNAs, lnc-CD164L2-1:1, lnc-CFP-1:1 and lnc-CLEC12B-1:1, all downregulated lncRNAs in tumor (Figure 1B and 1C), significantly correlated with the copy number variations of multiple locations on chromosomes 2, 11, 17, and 18 (Figure 5C–5E).

**Figure 3:**
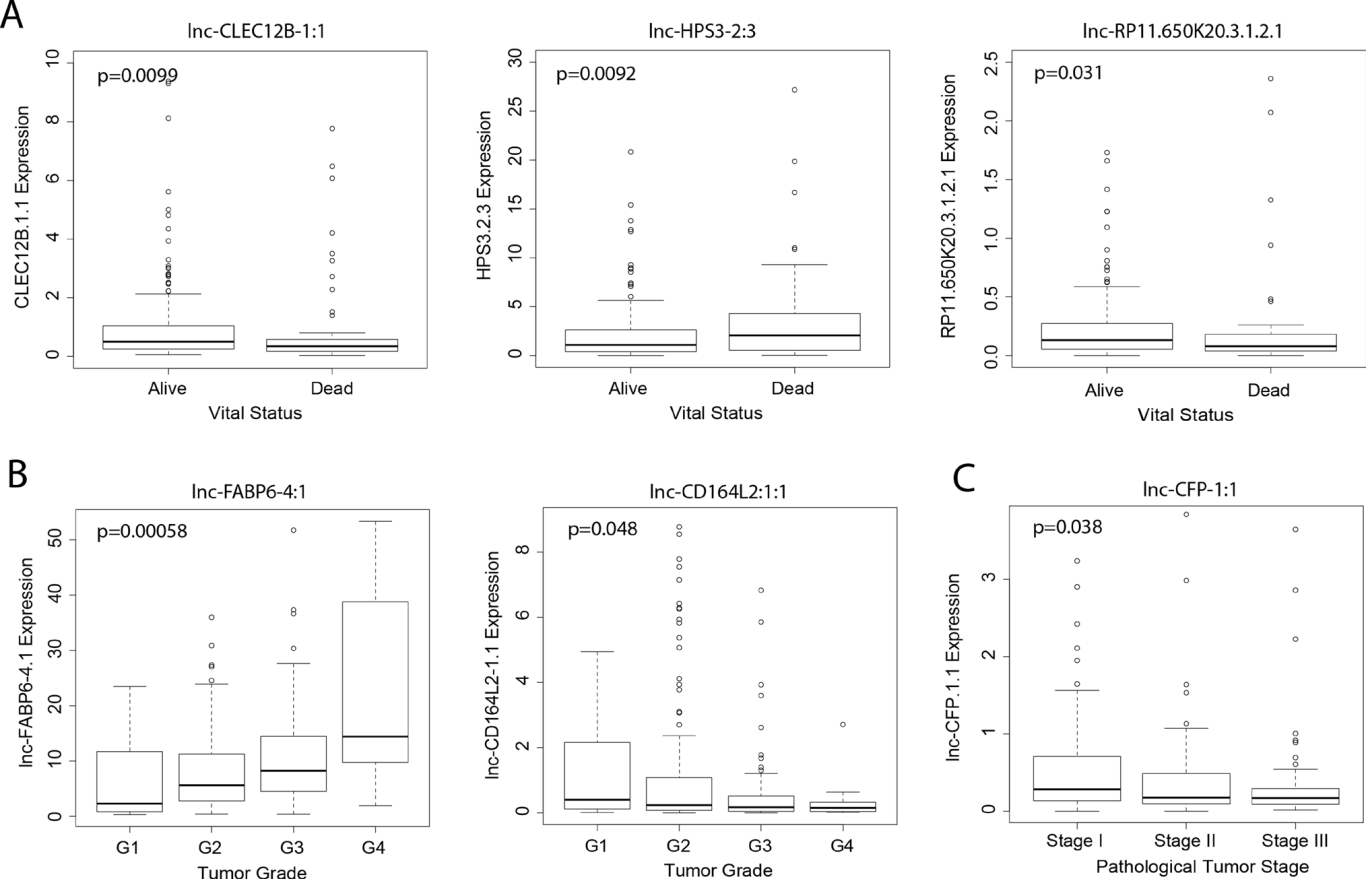
Association of lncRNA expression with clinical outcomes Boxplots correlating expression of lncRNAs (counts-per-million) to (**A**) vital status, (**B**) tumor grade, and (**C**) pathologic tumor stage (Wilcoxon rank sum, *p* < 0.05).

**Figure 4:**
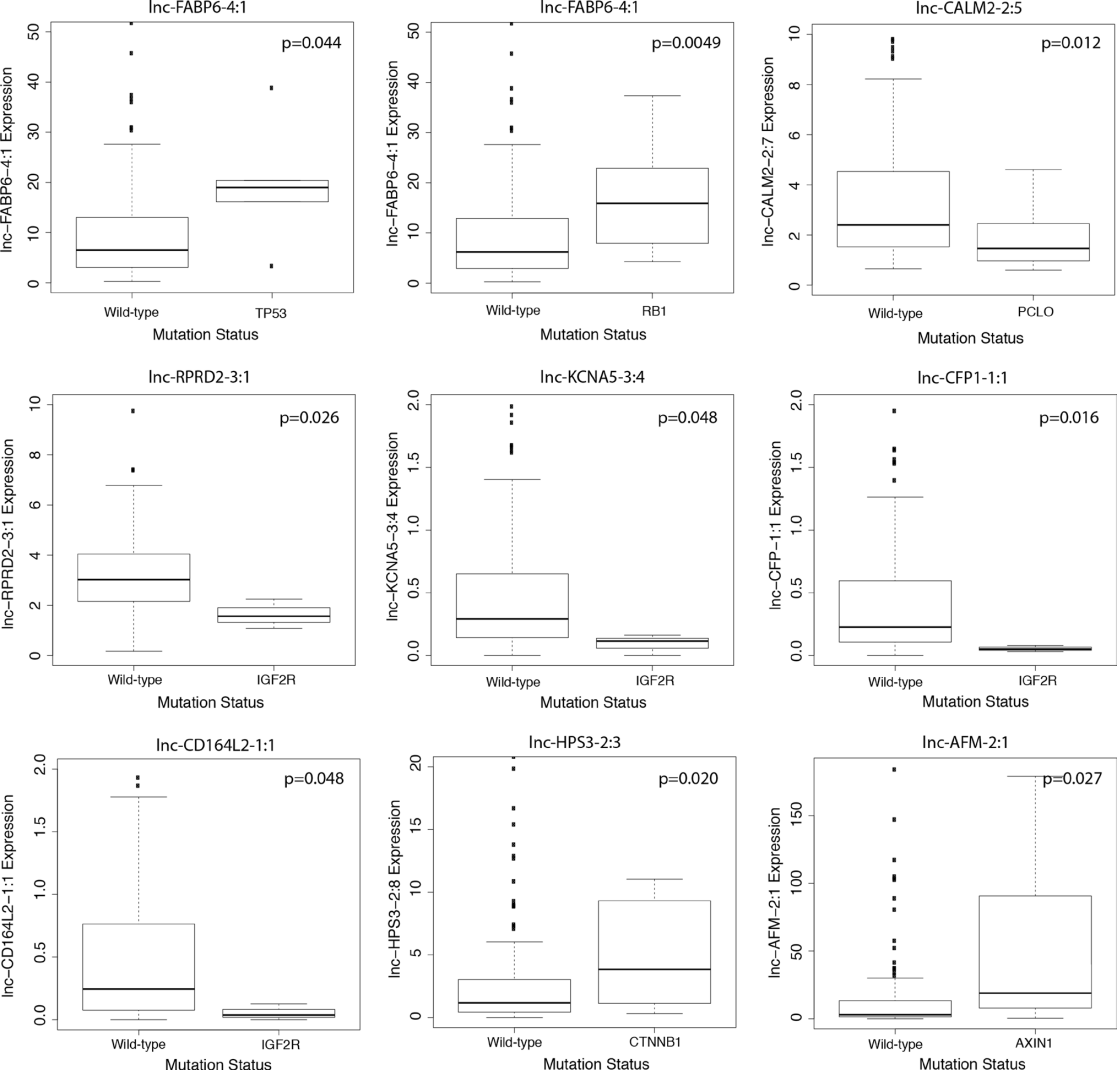
Correlation of lncRNA expression with gene mutations Boxplot showing significant correlation in expression level of lncRNAs (counts-per-million) and mutation status of frequently mutated genes in HCCs (Wilcoxon rank sum, *p* < 0.05).

**Figure 5:**
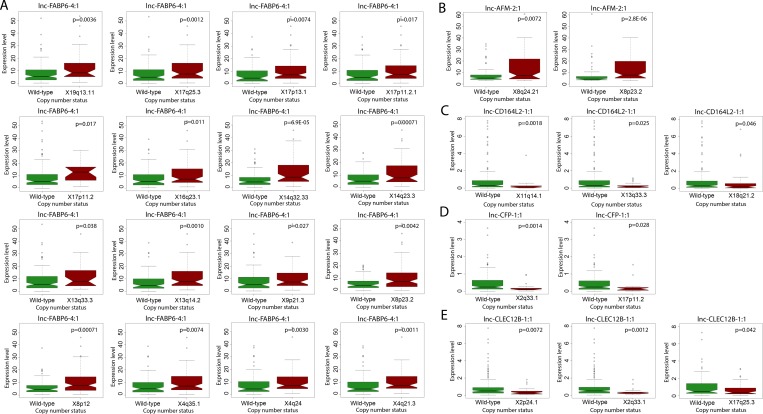
Correlation of lncRNA expression with copy number aberrations Boxplot showing significant correlation in copy number variation status and expression level (counts-per-million) of (**A**) lnc-FABP6-4:1, (**B**) lnc-AFM-2:1, (**C**) lnc-CD164L2-1:1, (**D**) lnc-CFP-1:1, and (**E**) lnc-CLEC12B-1:1 (Wilcoxon rank sum, *p* < 0.05).

### *In vitro* validation of alcohol-dysregulated lncRNAs in liver cell lines

**Figure 6:**
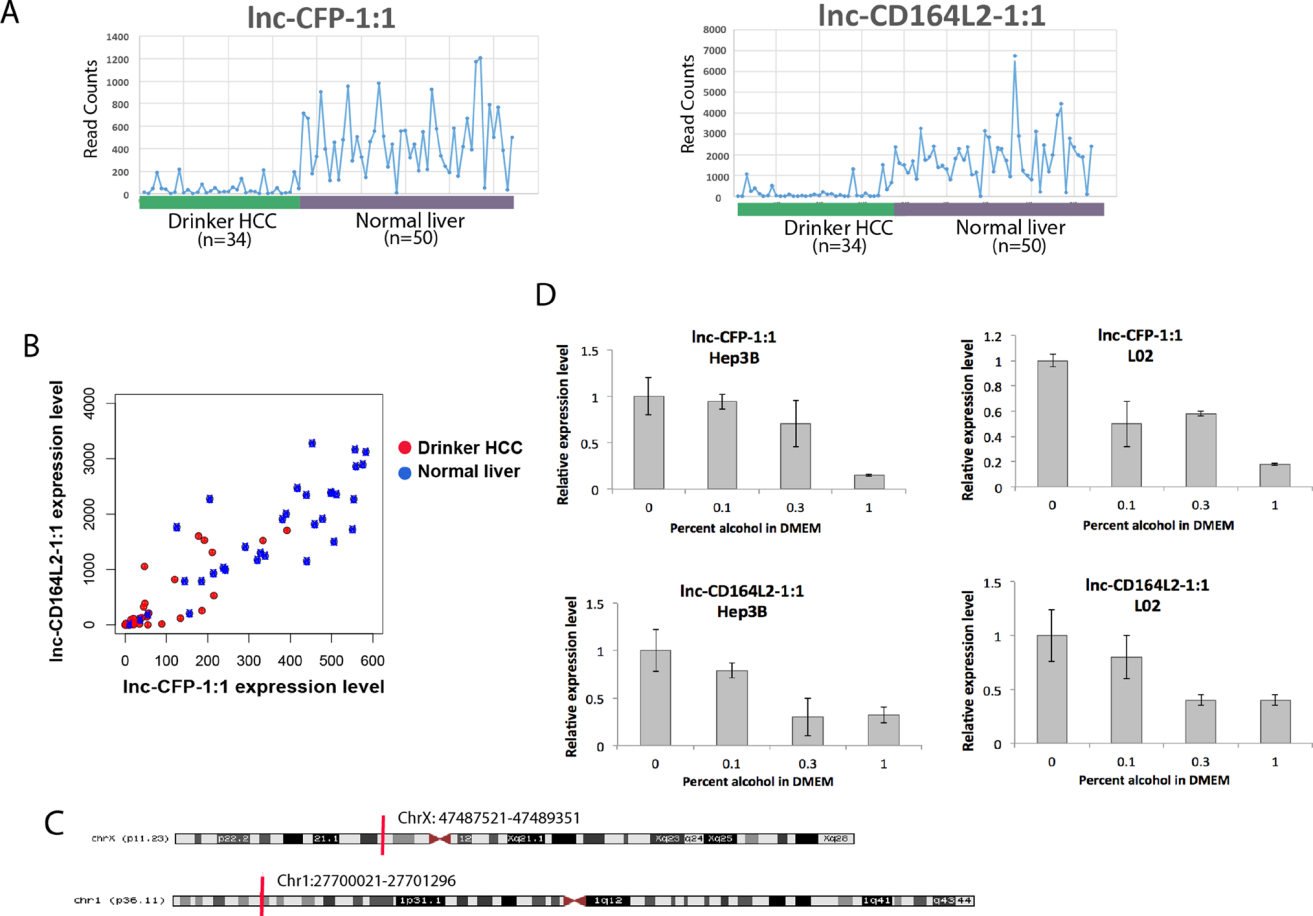
*In vitro* validation of alcohol-dysregulated lncRNAs in liver cell lines (**A**) Expression plot of lnc-CFP-1:1 and lnc-CD164L2-1:1 in HCC drinkers compared to normal liver tissues. (**B**) Scatter plots showing clusters of HCC drinkers and normal liver tissues with respect to expression levels of lnc-CFP-1:1 and lnc-CD164L2-1:1. (**C**) Loci of lnc-CFP-1:1 and lnc-CD164L2-1:1. (**D**) qRT-PCR verifies that 0.1%, 0.3% and 1% alcohol treatment downregulates lnc-CFP-1:1 and lnc-CD164L2-1:1 in L02 and Hep3B. All bar graphs are presented as the mean and error bars representing standard deviations.

We chose two lncRNAs, lnc-CFP-1:1 and lnc-CD164L2-1:1, for further *in vitro* validation for their strong correlations with both clinical outcomes and genomic alterations (Figures 2–5). Both of these lncRNAs are observed to be consistently downregulated in drinker HCC patients compared to normal livers (Figure 6A), with clusters of drinker HCC patients observed at low expressions for both lncRNAs (Figure 6B). lnc-CFP-1:1, a 569-nt transcript on chromosome X, and lnc-CD164L2-1:1, a 1,000-nt transcript on chromosome 1, have not been previously characterized. To validate their dysregulation due to alcohol, we treated the non-cancerous liver cell line L02 as well as the human hepatoma cell line Hep3B with 0.1% (17 mM), 0.3% (34 mM) and 1% (170 mM) ethanol for 7 days. Upon treatment with ethanol, both lncRNAs were observed to be significantly downregulated, in a dose-dependent fashion, in both cell lines, with lnc-CFP-1:1 reduced to less than 20% of its original expression at 170 mM alcohol.

## DISCUSSION

We investigated lncRNAs deregulated in alcohol-related HCC and HBV/HCV-related HCC to explore unique disease markers for different etiologies. lncRNAs have been documented as important functional molecules in the development of HBV/HCV-related HCC [17]. Our study identified 12, 328 differentially expressed lncRNAs between HBV-infected patient samples and normal samples. 20, 873 lncRNAs were differentially expressed between HCV positive samples and normal samples. 19, 194 lncRNAs were differentially expressed between samples positive for both HBV and HCV and normal samples. The 5, 456 lncRNAs that displayed significant differential expression in all three permutations above were most likely to be highly involved in hepatitis induction of HCC. Despite the large numbers of lncRNAs that are deregulated in patients with HBV/HCV-related HCC, only the mechanisms of a select few lncRNAs have been extensively studied [6, 17–20]. None of the most significantly dysregulated lncRNAs we found has been previously studied in HCC, suggesting that our current picture of lncRNA dysregulation in HCC is incomplete.

From our analysis, 53 lncRNAs were associated with alcohol-related HCC. We found no previous study that explored the relationship between lncRNAs and alcohol consumption in HCC. The reason may be that alcohol intake often indirectly leads to genetic dysregulation– through causing elevated acetaldehyde levels, accumulation of iron, chronic liver inflammation, or liver fibrosis/cirrhosis–while hepatitis viruses integrate viral DNA directly into the host genome to cause dysregulation of genetic mechanisms [21, 22]. For example, alcohol consumption can lead to liver fibrosis, which can progress to liver cirrhosis. Cirrhotic livers led to HCC in 90% of HCC cases [23]. Knowledge of dysregulated lncRNAs in alcohol-related HCC may lead to development of prevention or treatment strategies for alcoholic cirrhosis, thereby arresting disease progression to HCC.

Candidate lncRNAs from our analysis were correlated with patient survival and clinical variables. The expression of four lncRNAs differentially expressed in HCC versus normal samples– lnc-FABP6-4:1, lnc-CD164L2-1:1, lnc-CFP-1:1, and lnc-CLEC12B-1:1– correlated with survival rates in the direction of dysregulation. The expression of three lncRNAs dysregulated by alcohol drinking– lnc-CALM2-2:5, lnc-CALM2-2:7, and lnc-HPS3-2:3– also correlated with patient survival. For lncRNAs dysregulated by hepatitis, lnc-PKD2-2:2 and lnc-RP11-650K20.3.1-2:1 expressions correlated with patient survival. The expression of the majority of these lncRNAs also correlated with vital status, tumor grade, or pathological tumor stage.

We correlated the expression of survival-related lncRNAs with mutation status and copy number variation to gain an understanding of their putative involvement in dysregulated genetic pathways in HCC. lnc-FABP6-4:1 upregulation correlated with presence of TP53 and RB1 mutation. IGF2R mutation correlated with downregulation of lnc-CFP-1:1 and lnc-CD164L2-1:1. Lnc-CD164L2 has a gene locus resides in the intronic region of the protein coding gene CD164, which has been found to act as a metastasis promoter in prostate cancer [24]. CTNNB1 mutation correlated with upregulation of lnc-HPS3-2:3. TP53, a tumor suppressor gene, is the most commonly mutated gene in cancer, including in HCC; while CTNNB1 is the most commonly mutated proto-oncogene in HCC [25]. CTNNB1 is an integral element of the Wnt signaling pathway, which is activated in HCC and shown to be partly regulated by lncRNAs, such as lnc-DANCR [6]. RB1 is part of the RB1 tumor suppressing pathway, which is commonly inactivated in many cancers, including HCC [26]. IGF2R is also a tumor suppressor involved in multiple cancers and functions in HCC by inhibiting liver cell invasion [27].

To validate our correlations, we treated cells to different concentrations of alcohol *in vitro* and measured expression levels of lnc-CD164L2-1:1 and lnc-CFP-1:1, which correlated very well with patient survival and clinical variable. The decrease in their expression as alcohol concentration increases in both HCC and normal cell lines matches the direction of dysregulation in our statistical analysis and validates our hypothesis that alcohol dysregulates lncRNA expression.

No previous study, to the best of our knowledge, has explored the mechanism of possible synergism between the risk factors of hepatitis infection and alcohol intake. Studies from Italy, Taiwan, Japan, and the United States have found increased risk of HCC development when patients with HCV and HBV infections regularly drink alcohol, compared to non-drinking patients with hepatitis [28–31]. Further investigation of the mechanisms of lncRNAs we identified to be involved in alcohol-related HCC and HBV/HCV-related HCC may provide insight into this mechanism of synergism, knowledge that can lead to better identification of high-risk individuals and provide focus for development of preventative methods.

The early diagnosis of hepatocellular carcinoma is critical to more effective treatment and better prognosis since the few current treatment options for potentially curing HCC, such as liver transplantation, ablation, or resection, are effective only for the early-stages of HCC [32]. Ultrasonography can be used for early diagnosis but only has a sensitivity of 60–80%, despite a specificity of 94% [33]. Biomarkers such as serum alpha-fetoprotein (AFP) and des-gamma carboxyprothrombin (DCP) failed as useful diagnostic tools because of similar sensitivity as ultrasonography [1]. In contrast, studies utilizing combinations of circulating lncRNAs or lncRNA with other types of RNAs as diagnostic biomarkers for HCC achieved sensitivities of over 90% [34–37]. These studies suggested the usefulness of using lncRNAs as diagnostic markers, although the great majority of these studies investigated HCC cohort versus healthy liver cohort instead of taking specific risk factors into account [1]. Because different etiologies of HCC can result in dysregulation of different lncRNAs and disparate prognoses, our analysis of lncRNAs dysregulated in HCC induced by different risk factors can provide useful information about biomarkers present at the early stages of HCC.

Besides serving as diagnostic markers, lncRNAs can potentially be directly involved in the treatment of HCC. Direct delivery of tumor suppressing lncRNAs into liver cells or reducing expression of oncogenic lncRNAs through siRNA interference can be potential treatment strategies [1]. These options provide alternatives to the aforementioned invasive procedures for curing HCC. Furthermore, current treatment practices for HCC only consider the stage of disease, not its molecular etiology [38]. The use of lncRNAs for treatment may also lead to more personalized treatments of HCC for better therapeutic response.

## MATERIALS AND METHODS

### RNA-sequencing datasets and clinical data

RNA-sequencing datasets and clinical data for HCC patients and tumor-adjacent normal liver tissues were obtained on 8 July 2016 from The Cancer Genome Atlas (TCGA) (https://tcga-data.nci.nih.gov/tcga). 222 HCC patients and 50 tumor-adjacent normal liver tissues were retained for further analysis. These patients were sorted into four cohorts based on their clinical history on viral hepatitis infection and alcohol consumption: (1) HBV+ HCV- drinker (*n* = 34); (2) HBV+ HCV- non-drinker (*n* = 109); (3) HBV− HCV+ nondrinker (*n* = 32); and (4) HBV+ HCV+ nondrinker (*n* = 47).

### lncRNA differential expression analyses

lncRNA read counts were generated from RNA-sequencing datasets via BEDtools coverageBed (https://github.com/arq5x/bedtools2) [39] using lncRNA annotation files obtained from LNCipedia 3.0 (http://lncipedia.org/) [40], a database curating 113,438 lncRNA transcripts from sources including the Broad Institute, Ensembl, Gencode, Refseq, and NONCODE. The read count tables were imported into edgeR v3.0 (http://www.bioconductor.org/packages/release/bioc/html/edgeR.html) [41], and lowly expressed lncRNAs (counts-per-million <1 in more than one-half of samples) were filtered from the analysis. Following TMM normalization, pairwise designs were applied to identify significantly differentially expressed lncRNAs in (1) HBV+ HCV− drinker versus normal liver; (2) HBV+ HCV− non-drinker versus normal liver; (3) HBV+ HCV− drinker versus HBV+ HCV- non-drinker; (4) HBV− HCV+ non-drinker versus normal liver; and (5) HBV+ HCV+ non-drinker versus normal liver.

### Association of lncRNA expression with patient survival and other clinical outcomes

Survival analyses were performed using the Kaplan-Meier Model, with lncRNA expression in HCC tumors designated as a binary variable based on expression above or below the median. One patient with no clinical information was removed and 221 HCC patients were retained for the analysis. Employing the Kruskal-Wallis test and lncRNA expression values (counts-per-million), we investigated lncRNA association patient vital status, tumor grade and pathologic stages. HCC patients in all four cohorts were used in this analysis.

### Association of lncRNA expression with tumor mutations and copy number aberrations

Mutation calls for the HCC tumors were obtained from mutation annotation files (maf) generated by the Broad Institute GDAC Firehose on 5 September 2016. We focused our analysis on the 10 most frequently mutated genes in HCCs, as determined by Debuire *et al* [16]. Wilcoxon rank sum tests were employed to test for significant associations between lncRNA expression level (counts-per-million) and mutational status. Copy number variations for the TCGA tumors were obtained from the GISTIC2 pipeline in Broad GDAC Firehose on 6 July 2017. All significant (99% confidence) focal amplifications and deletions were analyzed for correlation to lncRNA expression level using Wilcoxon rank sum tests, followed by Benjamini-Hochberg correction of lncRNA *p*-values.

### Cell culture and treatments with ethanol

The non-cancerous liver cell line L02 and the human hepatoma cell line Hep3B were gifts from the Wang lab at University of Hong Kong. The cells were cultured in DMEM supplemented with 10% fetal bovine serum, 2% penicillin/streptomycin, and 2% L-glutamate (GIBCO) and maintained at 37°C in a humidified 5% CO2/95% air atmosphere. These cells were exposed to ethanol for 7 days. The doses used for ethanol treatment were 0.1 %, 0.3 %, and 1 % by volume (approximate concentrations 17 mM, 51 mM, and 170 mM, respectively). We chose the 0.1 % (17 mM) dose to represent social drinking habits, as 0.1 % is the blood alcohol level constituting legal intoxication in the U.S. [42]. The 0.3 % (51 mM) ethanol dose was used to simulate binge drinking habits, as it is representative of the blood alcohol levels of moderate to heavy drinkers [43]. The 1% (170 mM) ethanol dose, while potentially lethal in humans, was employed as an upper limit control. Treatment media was replaced every 24 hours with fresh media containing the stated ethanol concentration. The tissue culture plates were sealed with paraffin film to reduce evaporative loss of ethanol from the media.

### RNA isolation and cDNA synthesis

Upon completion of alcohol treatments, cells were harvested, and total cell lysates were collected. RNA was extracted using SurePrep RNA Isolation kit (Thermo Fisher Scientific, Inc.). Complementary DNA was synthesized according to the manufacturer’s protocol, using LncProfiler qPCR Array kit (catalogue no. RA900A-1; System Biosciences, Mountain View, CA, USA).

### qRT-PCR

qRT-PCRs were performed using FastStart Universal SYBR Green Master Mix (Roche Diagnostics) and run on a StepOnePlus Real-Time PCR System (Applied Biosystems). Results were analyzed using the ΔΔCt method and normalized to GAPDH expression. All primers were obtained from Bioneer and sequences are: GAACCTACT CTGTGCCAGCTC (lnc-CD164L2-1 forward), TTAC TCACCTGGCTCACCCT (lnc-CD164L2-1 reverse), ACCCCACGAAGGTAGAAGC (LNC-CFP-1:1 forward) and GAGGTTTCCACCCAATCCCA (LNC-CFP-1:1 reverse).
